# 16-Hydroxy-Lycopersene, a Polyisoprenoid Alcohol Isolated from *Tournefortia hirsutissima*, Inhibits Nitric Oxide Production in RAW 264.7 Cells and Induces Apoptosis in Hep3B Cells

**DOI:** 10.3390/molecules24132366

**Published:** 2019-06-26

**Authors:** Israel Hurtado-Díaz, Jessica Nayelli Sánchez-Carranza, Antonio Romero-Estrada, Leticia González-Maya, Judith González-Christen, Maribel Herrera-Ruiz, Laura Alvarez

**Affiliations:** 1 Centro de Investigaciones Químicas-IICBA, Universidad Autónoma del Estado de Morelos, Cuernavaca 62209, Morelos, Mexico; 2 Facultad de Farmacia, Universidad Autónoma del Estado de Morelos, Cuernavaca 62209, Morelos, Mexico; 3 Centro de Investigación Biomédica del Sur, Instituto Mexicano del Seguro Social, Xochitepec 62790, Morelos, Mexico

**Keywords:** 16-hydroxy-lycopersene, polyisoprenoid alcohols, *Tournefortia hirsutissima*, anti-inflammatory activity, apoptosis

## Abstract

Three polyisoprenoid alcohols were isolated from the leaves of *Tournefortia hirsutissima* by a bioassay-guided phytochemical investigation. The compounds were identified as 16-hydroxy-lycopersene (Compound **1**), (*Z*_8_,*E*_3_,ω)-dodecaprenol (Compound **2**) and (*Z*_9_,*E*_3_,ω)-tridecaprenol (Compound **3**). Compound **1**, an unusual polyisoprenoid, was characterized by 1D and 2D NMR. We also determined the absolute configuration at C-16 by the modified Mosher’s method. The in vitro antiproliferative and anti-inflammatory activities of the isolated compounds were evaluated. Among isolates, Compound **1** moderately inhibited the nitric oxide production in lipopolysaccharide (LPS)-stimulated RAW 264.7 cells. On the other hand, Compound **1** displayed selective antiproliferative activity against HeLa, PC3, HepG2 and Hep3B cancer cells and was less potent against IHH non-cancerous cells. Compound **1** in Hep3B cells showed significant inhibition of cell cycle progression increasing the sub-G_1_ phase, suggesting cell death. Acridine orange/ethidium bromide staining and Annexin V-FITC/PI staining demonstrated that cell death induced by Compound **1** in cells Hep3B was by apoptosis. Further study showed that apoptosis induced by Compound **1** in Hep3b cells is associated with the increase of the ratio of Bax/Bcl-2, and caspase 3/7 activation. These results suggest that Compound **1** induce apoptotic cell death by the mitochondrial pathway. To our knowledge, this is the first report about the presence of polyprenol Compounds **1**–**3** in *T. hirsutissima*, and the apoptotic and anti-inflammatory action of Compound **1**.

## 1. Introduction

Medicinal plants have served humans since prehistoric times to treat various ailments. More in depth studies are focused to identify compounds with specific cellular functions [1]. The molecular mechanism of action of such bioactive molecules may open new avenues to develop or improve novel therapeutic approaches to treat various ailments. For example, ursolic acid, a pentacyclic triterpene present in various plant species has multiple intracellular and extracellular targets on apoptosis, metastasis, angiogenesis and inflammatory processes [2]. The butanol extract from the bark of *Canarium tramdenum* showed antioxidant capacity and inhibited α-amylase and α-glucosidase. α- and β-amyrins were the most dominant constituents in this extract. These triterpenes highlighted the potentials of anti-inflammatory, anti-ulcer, anti-hyperlipidemic, anti-tumor, and hepatoprotective properties of *C. tramdenum* bark [3]. Salvinorin A from *Salvia divinorum* reduces inflammatory mediators in lipopolysaccharide (LPS)-stimulated macrophages and shows moderate anti-inflammatory effects in vivo. Additionally, salvinorin A inhibits intestinal contractility in the isolated guinea pig ileum [4]; *S*. *divinorum* has been used for recreational use and for the treatment of inflammatory disorders, rheumatism and headache. Paclitaxel (Taxol) is a tricyclic diterpenoid compound naturally produced in *Taxus brevifolia* and is one of the most successful and widely used natural anticancer drugs [5]. This diterpenoid is a microtubules stabilizing agent [6]. 

*Tournefortia hirsutissima* L. (Boraginaceae) is a shrub native to México, Central and South America, and the Antilles. Furthermore, it grows in the southern United States [7]. In México, it is known as “hierba rasposa” and people at the Sierra de Huautla, in the state of Morelos, use the leaves to wash wounds and for chafing, diarrhea and inflammation of the kidney [8]. In addition, in the state of Veracruz, México people use an infusion of the stems to treat diabetes [9]. On the other hand, in Trinidad and Tobago, it is used for hypertension, jaundice and diabetes [10]. The in vivo hypoglycemic and anti-hyperglycemic effects of this species were previously demonstrated [9,11]. In another work [12], a hybrid biomedical material, containing EtOH extract of *T. hirsutissima*, potentially useful in the healing of diabetic foot ulcerations was developed. 

Polyisoprenoid alcohols are known as acyclic polymers with more than five but less than 150 isoprene units per molecule. Their structural diversity mainly resides in the unsaturated bonds number, chain length of the carbon skeleton and configuration of the double bonds (*Z*/*E*). They are classified into types either all-*trans* (*E*) or mainly *cis* (*Z*) [13]. Polyisoprenoid alcohols diminish levels of serum cholesterol, prevent liver toxic injuries, and restore disrupted hepatic functions [14,15]. They also have antibacterial [16,17] and anti-inflammatory [18] activities, and their phosphate derivatives show apoptotic [19,20] activities.

In this study, we reported the isolation, purification and characterization of three polyisoprenoid alcohols (16-hydroxy-lycopersene (Compound **1**), (*Z*_8_,*E*_3_,ω)-dodecaprenol (Compound **2**) and (*Z*_9_,*E*_3_,ω)-tridecaprenol (Compound **3**)) from leaves of *T*. *hirsutissima* by bioassay-guided phytochemical investigation. We also evaluated the effect of the isolates on the nitric oxide production in LPS-stimulated RAW 264.7 cells. In addition, the antiproliferative effect of Compounds **1**–**3** against four human cell lines with high cancer incidence and mortality (PC3 (prostate), HeLa (cervical), Hep3B and HepG2 (hepatocellular)) was evaluated. Finally, we investigated the type of cell death induced by Compound **1** in Hep3B cells.

## 2. Results and Discussion

### 2.1. Pharmacological Activity of Tournefortia hirsutissima

With the aim to assess the pharmacological potential of *T. hirsutissima*, the n-hexane (Th-H), dichloromethane (Th-D) and hydroalcoholic (Th-HA) extracts were evaluated for their anti-inflammatory and antiproliferative properties. 

We assessed the anti-inflammatory activity of the extracts in the murine model of 12-*O*-tetradecanoylphorbol-13-acetate (TPA)-induced acute edema. Th-H, Th-D and Th-HA were evaluated in male ICR mice at 1 mg/ear dose. The results showed that Th-H had the best anti-edema effect with an inhibition of 78.0 ± 1.5%. A similar effect was found with positive control (indomethacin), which showed edema inhibition of 87.6 ± 0.4% at 1 mg/ear. Th-D and Th-HA were less active with inhibition values of 45.0 ± 6.7% and 28.7 ± 12.5%, respectively. In addition, the effect of the extracts on nitric oxide (NO) production in LPS-stimulated RAW 264.7 cells was evaluated. The cells treated with LPS resulted in a significant increase in NO production as 100% of the production. The positive control (indomethacin) inhibited NO production by 54.6 ± 8.9% at 30 µg/mL (*p* < 0.0001). In the presence of Th-H (75.7% ± 7.7% of inhibition at 30 µg/mL), the NO production was reduced in a concentration-dependent manner (Figure S2), being this extract more active than indomethacin, with an IC_50_ value of 11.2 µg/mL. The inhibitory effect of Th-H was not due to cytotoxicity, since it did not affect cell viability of RAW 264.7 cells up to a concentration of 60 µg/mL (Figure S3). In contrast, both Th-D (up to 60 µg/mL) and Th-HA (up to 120 µg/mL) did not show a significant inhibitory effect on NO production. Importantly, the vehicle DMSO (0.4%, *v*/*v*) did not show a significant decrease in both the cell viability and NO production. 

The antiproliferative activity of the extracts was evaluated against Hep3B and HepG2 human cancer cell lines by MTS assay. We also included immortalized human hepatocyte cell line (IHH) as a control of non-cancerous cells. Results indicated that the *n*-hexane extract (Th-H) showed the best antiproliferative effect with IC_50_ values of 32.7 ± 0.9 against Hep3B, and 21.6 ± 1.9 µg/mL against HepG2. These cells were slightly less sensitive against Th-HA (IC_50_ = 36.1 ± 1.5 µg/mL in Hep3B and IC_50_ = 31.0 ± 1.4 µg/mL in HepG2). Th-D was the least cytotoxic, showing an IC_50_ value of 33.6 ± 2.2 µg/mL against HepG2, and a negligible cytotoxicity in Hep3B cells with an inhibitory effect in cell proliferation < 50% to 100 µg/mL. The three extracts had a negligible antiproliferative effect in the IHH cell line, suggesting that these extracts contain compounds with selectivity towards cancer cells.

Due to the Th-H extract showed the best effect in both anti-inflammatory and antiproliferative models, this extract was fractionated monitoring this process by evaluating the inhibitory effect of the different fractions on NO production in LPS-stimulated RAW 264.7 cells. Open column chromatography of the Th-H extract afforded six fractions F1–F6. Due to their higher abundance, F4 and F5 were further evaluated. Fraction F4 inhibited the NO production with an IC_50_ value of 36.5 µg/mL. The inhibitory effect of F5 was not determined, since this fraction affected the cell viability from 15 µg/mL. Thus, F4 was fractionated to yield four subfractions F4-1–F4-4 and the pharmacological evaluation showed that F4-2 was the most active with 50.5 ± 12% of inhibition at 25 µg/mL (*p* < 0.0001). Fraction F4-4 had a lower inhibitory effect with 46.3 ± 16.6% at 100 µg/mL and F4-1 did not inhibited the NO production. Fraction F4-3 could not be evaluate, since its solubility was poor. Then, the most active subfraction (F4-2) was fractionated to obtain F4-2-1 and F4-2-2 fractions.

### 2.2. Purification and Characterization of Polyisoprenoid Alcohols

**Figure 1:**
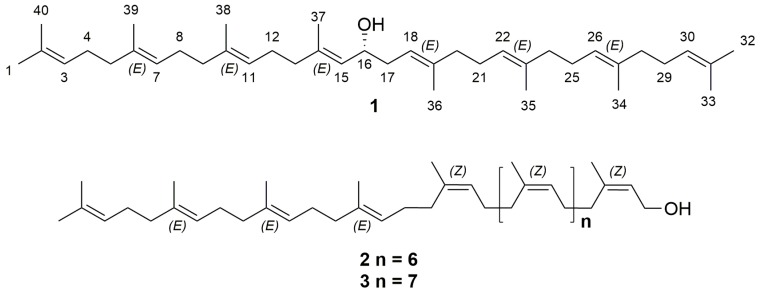
Chemical structures of the polyisoprenoid alcohol Compounds **1**–**3**.

F4-2-1 was subjected to GC/MS analysis, indicating that bis (2-ethylhexyl) phthalate was the only major component in this fraction. F4-2-2 was subjected to a reversed-phase preparative HPLC purification to yield the polyisoprenoid alcohols 16-hydroxy-lycopersene (**1**), (*Z*_8_,*E*_3_,ω)-dodecaprenol (**2**) and (*Z*_9_,*E*_3_,ω)-tridecaprenol (**3**) (Figure 1). 

**Figure 2:**
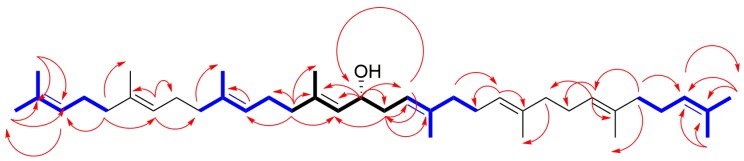
Key correlations in the COSY (black colored), TOCSY (blue colored) and HMBC (red arrows) spectra used for the characterization of Compound **1**.

Compound **1** was obtained as a light-yellow oil and its molecular formula was determined as C_40_H_66_O by high resolution ESIQTOFMS. (*m*/*z* 563.5238 [M + H]^+^, calculated for C_40_H_67_O, 563.5186). The ^1^H NMR data of **1** revealed signals for eight olefinic methines (between δ_H_ 5.37 and 5.23 (8H, H-3, -7, -11, -15, -18, -22, -26 and -30)), a hydroxylated methine (δ_H_ 4.39 (1H, H-16)) and twenty-six methylene protons (δ_H_ 2.04 (2H, H-13), 2.26 and 2.40 (2H, H-17), and between 2.23 and 2.07 (22H)). In addition, ten methyl groups were located at δ_H_ 1.57 (6H, H-33 and -40), 1.59 (3H, H-37), 1.60 (6H, H-35 and -38), 1.61 (3H, H-36), 1.62 (6H, H-34 and -39) and 1.69 (6H, H-1 and -32). The DEPTQ NMR spectrum exhibited signals of ^13^C for eight trisubstituted double bonds between δ_C_ 130 and 120 (C-3, -7, -11, -15, -18, -22, -26 and -30) and between δ_C_ 138 and 131 (C-2, -6, -10, -14, -19, -23, -27 and -31). The signal at δ_C_ 68.62 (C-16) corroborated the presence of a methine carbinol group. The configuration of the internal prenyl residues resulted to be *E* according to the chemical shifts of methyl carbons C-34, -35, -36, -37, -38 and -39 between δ_C_ 16.76 and 16.17 [21]. The COSY correlations permitted the assignation of the resonances at δ_H_ 5.36 (H-15), 2.40 and 2.26 (H-17) by crosspeaks with δ_H_ 4.39 (H-16). Noteworthy, the correlation H-16/H-17 establishes a connectivity head to head of the two prenyl residues. Regarding the resonance for H-15 at δ_H_ 5.36, this showed crosspeaks with the resonances at δ_H_ 2.04 (H-13) and 1.59 (H-37), while the resonances for H-17 at δ_H_ 2.26 and 2.40 showed a crosspeak with H-18 (δ_H_ 5.34). Analysis of the TOCSY spectrum allowed us to establish the unequivocal assignment for the protons H-12 (δ_H_ 2.19 to 2.15), H-11 (δ_H_ 5.27), H-9 (δ_H_ 2.12 to 2.08), and the methyl protons H-38 (δ_H_ 1.60), from the first prenyl chain, through their correlations with H-13 (δ_H_ 2.04). By the same way, the signals for H-17 (δ_H_ 2.26 and 2.40) were the starting point to delineate the spin network that identified protons H-20 (δ_H_ 2.11 to 2.09), H-21 (δ_H_ 2.21 to 2.16) and H-36 (δ_H_ 1.61) of the second prenyl chain. In addition, with the TOCSY spectrum, we identified the terminal methyl protons (H-1 and H-32 (*Z,* δ_H_ 1.69), and H-33 and H-40 (*E*, δ_H_ 1.57)) by the crosspeak between δ_H_ 1.69 (the *Z*) and 1.57 (the *E*). The resonances for the following couples of protons H-3 with H-30, H-4 with H-29 and H-5 with H-28 were also overlapped and showed crosspeaks with H-1, H-32, H-33 and H-40, respectively. The HSQC spectrum gave the ^1^*J*_H-C_ correlations. The HMBC spectrum was used to assign the quaternary carbons and to corroborate the previously established assignations. In addition, with the aid of HMBC and HSQC spectra was possible assign the remaining resonances (see, Figure 2 for key correlations). The chemical shifts of ^1^H and ^13^C for **1** are shown in Table 1. 

**Figure 3:**
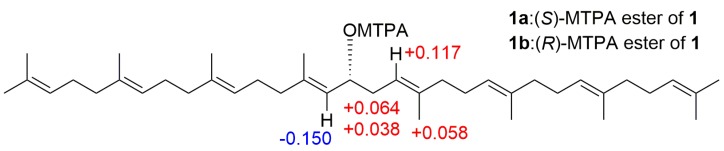
Δδ_H_ (Δδ_H_ = δ*_S_* − δ*_R_*) values obtained of (*S*)- and (*R*)-MTPA esters (**1a** and **1b**).

Compound **1** has only been isolated and characterized from the aquatic plant *Myriophyllum verticillatum* [22], but its absolute configuration was not assigned. Therefore, the absolute configuration was determined by the modified Mosher’s method. Based on the Δδ_H_ values of the (*S*)- and (*R*)-MTPA esters (Compounds **1a** and **1b**) obtained from Compound **1** Figure 3), the absolute configuration of C-16 was assigned as *R*. According to the above data, the structure of 16-hydroxy-lycopersene (Compound **1**) was characterized as (6*E*,10*E*,14*E*,18*E*,22*E*,26*E*)-2,6,10,14,19,23,27,31-octamethyldotriaconta-2,6,10,14,18,22,26,30-octaen-16*R*-ol. 

The other polyisoprenoid were identified by the comparison of their ^1^H NMR data with those reported in the literature for (*Z*_8_,*E*_3_,ω)-dodecaprenol (Compound **2**) [22,23] and (*Z*_9_,*E*_3_,ω)-tridecaprenol (Compound **3**) [22]. Noteworthy, Compounds **1**–**3** were isolated from *T*. *hirsutissima* for the first time in this work. 

### 2.3. Effect of Compounds on NO Production in LPS-Stimulated RAW 264.7 Cells

**Figure 4:**
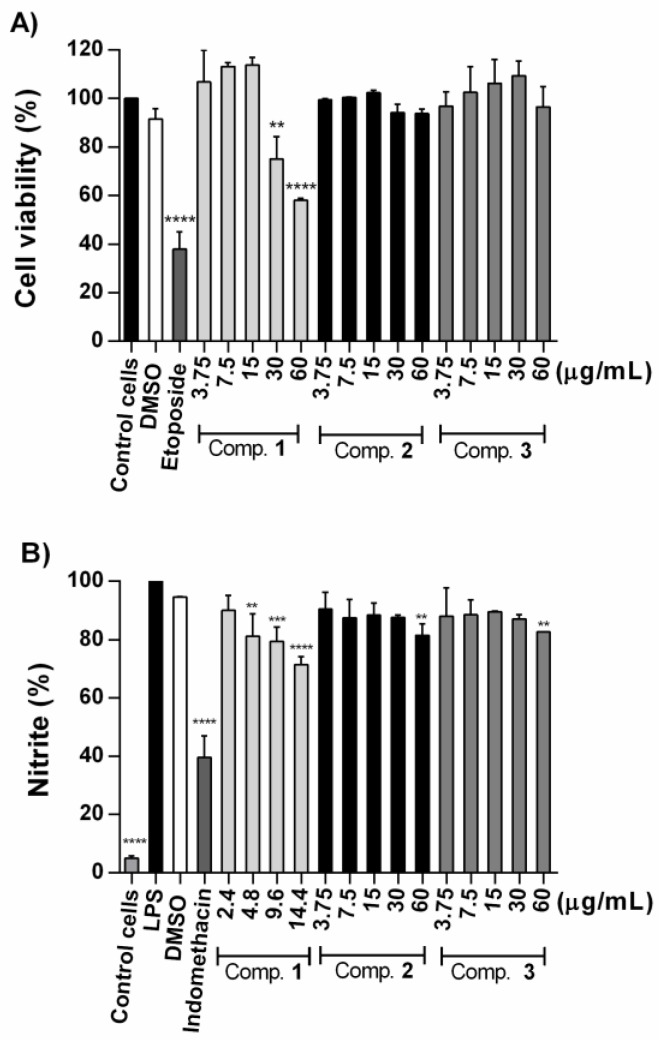
(**A**) Effect of Compounds **1**–**3** on cell viability of RAW 264.7 cells. Cells were incubated for 20 h with Compounds **1**–**3** (3.75–60 µg/mL), then were incubated with MTS for 4 h. Cell viability is expressed in percentage. (**B**) Effect of Compounds **1**–**3** on nitric oxide (NO) production in LPS-stimulated RAW 264.7 cells. Cells were treated with Compounds **1** (2.4–14.4 µg/mL), **2** and **3** (3.75–60 µg/mL), DMSO (0.4%, *v*/*v*) or indomethacin (30 µg/mL) 2 h before stimulation with LPS (1.0 µg/mL) for 21 h. Nitrite was determined by Griess method and is expressed in percentage. All data represent the mean ± S.D. of at least three independent experiments each by triplicate. Statistical significance was determined by one-way ANOVA followed by Dunnett’s test. * *p* < 0.05, ** *p* < 0.01, *** *p* < 0.001 and **** *p* < 0.0001 compared with control cells in (**A**) and group treated LPS in (**B**).

Compounds **1**–**3** were evaluated for their inhibition in the NO production in LPS-stimulated RAW 264.7 cells. The results showed that Compound **1** decreased the cell viability at 30 and 60 µg/mL (Figure 4A). Therefore, Compound **1** was evaluated below its highest non-cytoxic concentration. Compound **1** exhibited moderate and concentration-dependent inhibition, at 14.4 µg/mL (25.6 µM) inhibited NO production by 28.55 ± 1.81%. Compounds **2** and **3** showed negligible inhibitory activity at the highest evaluated concentration (60 µg/mL) (Figure 4B). This is the second report about the inhibitory effect of a polyisoprenoid alcohol in LPS-stimulated RAW 264.7 cells. Recently, was demonstrated that solanesol (40 µM), another all-trans polyprenol, suppresses the production of pro-inflammatory cytokines via heme oxygenase-1 induction [18].

### 2.4. Antiproliferative Activity of Compounds 

Compounds **1**–**3** were tested against four cancer cell lines (HeLa, PC3, HepG2 and Hep3B), and the IHH no-cancerous cell line. Results of maximal inhibitory concentration (IC_50_) are summarized in Table 2. Compound **1** had the best antiproliferative effect on all cancer cell lines, HeLa, PC3, HepG2 and Hep3B with IC_50_ values of 37.3 ± 3.5, 32.0 ± 4.4, 26.6 ± 3.5 and 21.3 ± 3.5 µM, respectively. Noteworthy, Compound **1** was selective toward cancer cells, due to that it was less cytotoxic against not-cancerous IHH cells with an IC_50_ value of 88.8 ± 8.9 µM. Compounds **2** and **3** exhibited negligible cytotoxicity against all the cancer cell lines and IHH cells (IC_50_ values between 50.3 ± 5.9 and 95.7 ± 12.0 µM). 

### 2.5. Apoptosis Induced by Compound ***1***

Since Compound **1** had the highest antiproliferative activity against all cancer cells, we examined its effect on the cell cycle progression by flow cytometry (Figure S15). We used paclitaxel (PTX) as a control of arrest of G_2_/M phase. Treatment with the IC_50_ values of Compound **1** increased the population in sub-G_1_ phase in Hep3B (from 0.0 to 18.5%, Figure S15A), HepG2 (from 1.5 to 14.8%, Figure S15B) and PC3 (from 0.9 to 19.0%, Figure S15C) cells. The increase in the sub-G_1_ phase of the cell cycle with respect to the control cells is associated with a lower DNA content, suggesting cell death. Some investigations support a correlation between apoptosis induced by chemical compounds and the increase in the sub-G1 phase of the cell cycle [24,25,26]. Hence, we proceeded to characterize the type of cell death in the Hep3B cells induced by Compound **1**, since it was the most sensitive cell line according to the IC_50_.

**Figure 5:**
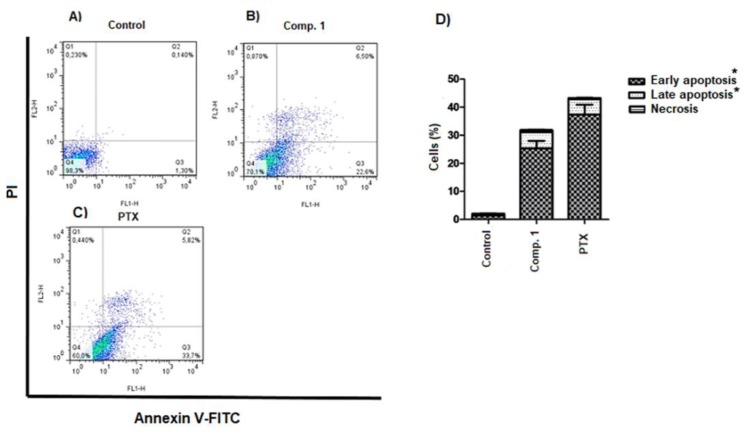
16-hydroxy-lycopersene (Compound **1**) induces apoptosis in Hep3B cells. Cell death analysis in Hep3B cells by flow cytometry using Annexin V-FITC/PI double staining. Early apoptotic cells (annexin V-positive and PI-negative; lower right quadrants), late apoptotic cells (annexin V-positive and PI-positive; upper right quadrants), and necrotic cells (annexin V-negative and PI-positive; upper left quadrants). (**A**) Untreated cells, (**B**) treated with Compound **1** at 21.3 µM (12 µg/mL) for 72 h and (**C**) treated with PTX 20 nM. (**D**) % of cells. The data are expressed as the means ± S.E.M. of three independent experiments. Statistical significance was determined by one-way ANOVA followed by Dunnett’s test. * *p* < 0.05 compared to the control.

First, we determined whether Compound **1** induced cell death was caused by apoptosis or necrosis. Phosphatidylserine externalization, a hallmark of early phase apoptosis, was analyzed using Annexin V-FITC/PI double staining and flow cytometry (Figure 5). The results indicated that the percentage of early phase apoptosis cells increased 22.6% after treatment with Compound **1** (Figure 5B,D) and 38% with PTX (positive control of apoptosis), demonstrating that Compound **1** induced Hep3B cell apoptosis. 

**Figure 6:**
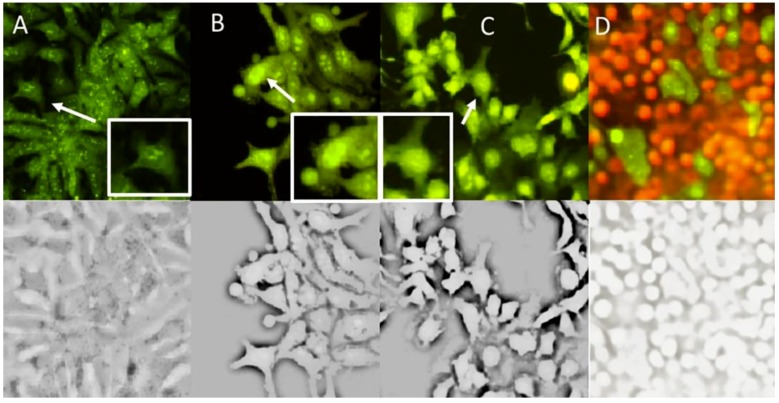
Analysis of cell death in Hep3B cells by using epifluorescence microscopy. (**A**) Negative control (cells without treatment). (**B**) Treatment with Compound **1** to 21.3 µM (12 µg/mL) for 72 h. (**C**) H_2_O_2_ apoptosis positive control. (**D**) Necrosis control.

To further verify this apoptotic cell death induced by Compound **1** in Hep3B cells, we analyzed morphological changes. Hep3B cells were treated with Compound **1** and observed by fluorescence microscopy of acridine orange/ethidium bromide. The treatment with Compound **1** (Figure 6B) showed apoptotic bodies formation and clear condensation of chromatin bright green nuclei (white arrow), characteristic changes of necrosis were not observed. These morphological changes were similar than those induced by H_2_O_2_ used as apoptosis positive control (Figure 6C). Therefore, this study also supports that Compound **1** induces apoptosis in Hep3B cells. 

**Figure 7:**
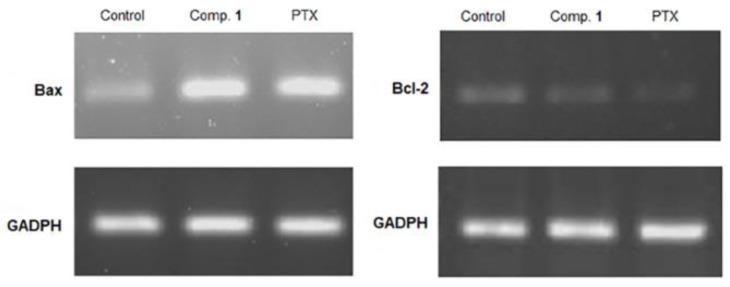
Effect of Compound **1** (21.3 µM) for 72 h, on mRNA expression levels of Bcl-2 and Bax in Hep3B cell line. Paclitaxel (PTX) (20 nM) was used as positive control. GAPDH was used as an internal control.

Caspases play a key role in the initiation and execution of apoptosis. It is well documented that activating caspase-9, cause the activation of effector caspases (-3, -6 and -7) in the apoptosis of intrinsic pathway; moreover, an increase of the pro-apoptotic Bax expression and down-regulation of the anti-apoptotic Bcl-2 is observed [27]. Therefore, the effect of Compound **1**, on the levels of Bax/Bcl2 and caspase 3/7 activation in Hep3B cells was determined. The results showed the inhibition of Bcl-2 and increased Bax transcripts (Figure 7), thus increasing the Bax/Bcl-2 ratio when compared to the non-treated control. 

**Figure 8:**
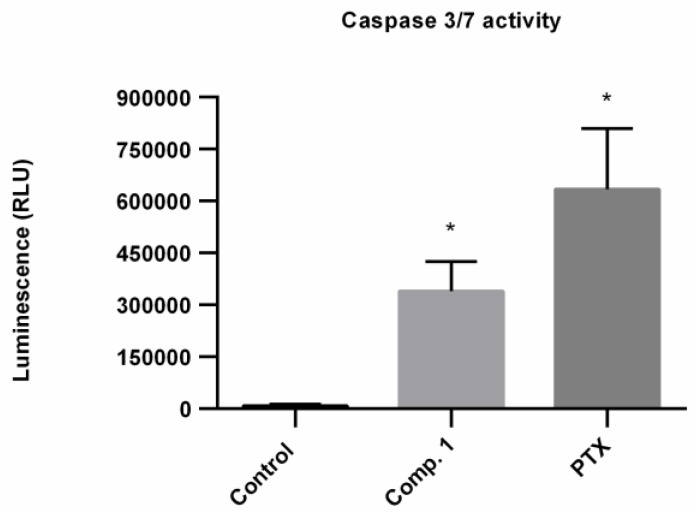
Caspase 3/7 activation after treatment with Compound **1** at 21.3 µM (12 µg/mL) for 72 h and PTX (20 nM) positive control on Hep3B cells. * *p* < 0.05 compared with the control cells group.

In addition, a clear statistically significant increase in caspase 3/7 activation was observed (Figure 8) which indicated that the antiproliferative activity of polyprenol **1** is due to apoptotic cell death. Some phosphated analogs of Compound **1** have shown apoptotic activity, too. Dolichyl phosphate (8 µg/mL) induced apoptosis in U937 cells by the MAP kinase cascade [20]. Dihydroheptaprenyl and dihydrodecaprenyl phosphates (6 µM) induced apoptosis in U937 cells was mediated by caspase-3-like (CPP32-like) activation but not by caspase-1-like (ICE-like) activation [19]. Our results suggest that Compound **1** cause cell death in Hep3B cells by the mitochondrial apoptotic pathway. It is important to mention that this is the first report about the apoptotic activity of Compound **1**. This plant species is widely used by the people of Morelos State in México, principally for the treatment of affections related with inflammation. According with the results here presented, the obtaining of a natural remedy applied in the clinic could serve also as a preventive to cancer development. 

## 3. Materials and Methods

### 3.1. General Experimental Procedures

NMR spectra of 1D and 2D were conducted on a Bruker AVANCE III HD 500 MHz spectrometer (Billerica, MA, USA). The chemical shifts are given in ppm (δ) and the spectra were referenced using the non-deuterated residual solvent signal. The ESIQTOFMS spectrum was obtained with a 1290 Infinity II liquid chromatograph Agilent coupled with an Agilent 6545 Accurate-Mass Quadrupole of Time of Flight equipped with an Agilent Jet Stream dual electrospray ionization source (Santa Clara, CA, USA). The GC-MS system consisted of an Agilent 6890 gas chromatograph and an Agilent 5970 mass selective detector (Santa Clara, CA, USA). in the electron-ionization mode at 70 eV with an HP-5 column (25 m × 0.2 mm, Hewlett Packard, Santa Clara, CA, USA). The analytical HPLC system used to analyze F4-2-2 consisted of an Agilent Technologies 1260 Infinity HPLC instrument (G1311B quaternary pump, G1329A auto-injector, G1316A thermostatted column compartment and G1315D diode array detector (DAD), Santa Clara, CA, USA) equipped with a Merck column (Chromolith^®^, Performance RP-18e, 100 × 4.6 mm, Darmstadt, HE, Germany). The isolation of Compounds **1**–**3** was performed using an Agilent Technologies 1260 Infinity preparative HPLC instrument (G1361A binary pump, manual injector, G1315D diode array detector] equipped with a Phenomenex column (Kinetex^®^ EVO, C-18, 150 × 10.0 mm, 5 μm, 100 Å, Torrance, CA, USA). Heptane, isopropanol (iPrOH) and methanol (MeOH) of HPLC grade were purchased from J.T Baker (Phillipsburg, NJ, USA). Water was milli-Q grade. Optical rotation was measured on a Perkin-Elmer 341 digital polarimeter (Perkin Elmer, Waltham, MA, USA). Merck silica gel (70–230, 230–400 mesh) was used for column chromatography (CC) and TLC plates (silica gel 60 F254, Merck, Darmstadt, HE, Germany) were used for analyzing the fractions which were visualized with a solution of Ce(SO_4_)_2_ 2(NH_4_)2SO_4_ 2H_2_O followed by heating. 

### 3.2. Plant Material

Aerial parts of *Tournefortia hirsutissima* L. were collected in Yautepec, in the state of Morelos, México in December 2015 by the MSc Israel Hurtado Díaz and identified by the Biol. Gabriel Flores Franco. A voucher specimen (no. 33912) was deposited at the HUMO Herbarium of Centro de Investigación en Biodiversidad y Conservación (CIByC) from the Universidad Autónoma del Estado de Morelos.

### 3.3. Preparation of Extracts

The dried leaves of *T*. *hirsutissima* (480 g) were powdered and extracted at room temperature with n-hexane, dichloromethane and H_2_O: CH_3_OH (15:85, *v*/*v*) in increasing order of the polarity (4 × 2000 mL, 24 h each). The extracts were filtered and concentrated in a rotary evaporator at 40 °C (organic extracts) and 60 °C (hydroalcoholic extract), resulting in the hexane extract Th-H (yield. 1.50%), the dichloromethane extract Th-D (yield. 0.63%) and the hydroalcoholic extract Th-HA (yield. 4.08%).

### 3.4. Isolation and Purification of Polyisoprenoid Alcohol Compounds ***1***–***3***

Th-H (4.9 g) was chromatographed by open column chromatography on silica gel (193 g, 70–230 mesh) eluting with a gradient of n-hexane-acetone (from 100:00 to 00:100, *v*/*v*) to yield six fractions (F1 (100:00); 72 mg, F2 (100:00); 22 mg, F3 (99:01); 25 mg, F4 (90:10); 2968 mg, F5 (85:15); 823 mg and F6 (00:100); 123 mg) grouped by TLC. F4 (2.5 g) was chromatographed on silica gel (100 g, 230–400 mesh) eluting with a gradient of n-hexane-acetone (from 96:04 to 88:12, *v*/*v*) to yield four subfractions (F4-1 (96:04); 53.3 mg), F4-2 (96:04); 1086 mg, F4-3 (92:08); 527 mg and F4-4 (88:12); 623 mg] grouped by TLC. F4-2 (1086 mg) was fractionated by CC on silica gel (40 g, 230–400 mesh) eluting with a isocratic system of n-hexane-acetone (97:03, *v*/*v*) to yield F4-2-1; 140.0 mg and F4-2-2; 940.0 mg. F4-2-2 was solubilized in iPrOH : MeOH : heptane (60:20:20, *v*/*v*) at the concentration of 1.5 mg/mL and the solution was filtered through a 0.45 µm filter. Subsequently, this solution was subjected to analytical HPLC analysis. The chromatographic conditions that allowed the separation of the components were as follow: Merck column (Performance RP-18e, 100 × 4.6 mm); injection volume of 7.0 µL; flow rate of 2.0 mL/min for 7.5 min; elution was a gradient of iPrOH:MeOH:H_2_O (from 30:67:03 to 30:70:00, *v*/*v*); temperature of 38 °C; detection at λ 210 nm. Three main peaks were identified in the chromatogram at retention times of 1.90, 4.14 and 5.43 min (Figure S4). The above method with minor modifications was used to yield Compounds **1**–**3** by using preparative HPLC. We used a Phenomenex column (C-18, 150 × 10.0 mm, 5 μm, 100 Å); injection volume of 200 µL of F4-2-2 (50 mg/mL); flow rate of 6.0 mL/min for 10.0 min; elution was isocratic with 30% A:70% B [A (100% iPrOH) and B (99% MeOH:1% H_2_O)]; room temperature; detection at λ 210 nm. The collecting of the peaks at retention times of 2.25, 4.52 and 6.03 min of five repetitive injections resulted in the isolation of Compound **1** (9.0 mg), Compound **2** (17.5 mg) and Compound **3** (10.7 mg), respectively. 

(6*E*,10*E*,14*E*,18*E*,22*E*,26*E*)-2,6,10,14,19,23,27,31-octamethyldotriaconta-2,6,10,14,18,22,26,30-octaen-16*R*-ol (16*R*-hydroxy-lycopersene (Compound **1**)). Light yellow oil; [α]^24^_D_ + 5.3 (c 0.8, CHCl_3_); ^1^H and ^13^C NMR data are presented in Table Compound **1**; ESIQTOFMS *m/z* 563.5238 [M + H]^+^ calculated for C_40_H_67_O, *m/z* 563.5186.

(*Z*_8_,*E*_3_,ω)-dodecaprenol (Compound **2**). Light yellow oil; ^1^H NMR (500 MHz, CDCl_3_) δ_H_: 5.44 (1H, H-2), 5.17–5.06 (11H), 4.09 (2H, H-1), 2.13–1.94 (44H), 1.74 (3H, CH_3_ in *Z*), 1.68 (24H, 8CH_3_ in *Z*), 1.60 y 1.61 (12H, 4CH_3_ in *E*). These data match those in the literature [22,23].

(*Z*_9_,*E*_3_,ω)-tridecaprenol (Compound **3**). Light yellow oil; ^1^H NMR (500 MHz, CDCl_3_) δ_H_: 5.44 (1H, H-2), 5.15–5.07 (12H), 4.09 (2H, H-1), 2.11–1.95 (48H), 1.74 (3H, CH_3_ in *Z*), 1.68 (27H, 9CH_3_ in *Z*), 1.60 y 1.61 (12H, 4CH_3_ in *E*). These data match those in the literature [22].

### 3.5. Preparation of (S)- and (R)-MTPA Esters of Compound ***1*** (***1a*** and ***1b***)

To a solution of Compound **1** (2.5 mg (1 eq)) in deuterated pyridine (0.1 mL) was added (*R*)-MTPA chloride (10.0 µL (12 eq)) under atmosphere of nitrogen. The mixture was stirred at room temperature for overnight to obtain the (*S*)-MTPA ester (**1a**). The same procedure was used to prepare the (*R*)-MTPA ester (**1b**) with (*S*)-MTPA chloride. (*R*)-MTPA and (*S*)-MTPA chlorides were purchase from Sigma Aldrich (St. Louis, MO, USA).

(*S*)-MTPA ester of 1 (**1a**). ^1^H NMR data (500 MHz, pyridine-*d*_5_) δ_H_ 5.321 (1H, H-15), 2.657 and 2.470 (2H, H-17), 5.348 (1H, H-18), 1.676 (3H, H-36).

(*R*)-MTPA ester of 1 (**1b**). ^1^H NMR data (500 MHz, pyridine-*d*_5_) δ_H_ 5.471 (1H, H-15), 2.593 and 2.432 (2H, H-17), 5.231 (1H, H-18), 1.618 (3H, H-36).

### 3.6. Cell Viability of the RAW 264.7 Cells 

RAW 264.7 cells (murine macrophage cell line) were purchased from ATCC (Georgetown, Washington, DC, USA ) and cultured in Advanced DMEM/F12 medium (Gibco, Waltham, MA USA) supplemented with 1% GlutaMAX (Gibco) and 3.5% inactivated FBS (Gibco), without antibiotics at 37 °C in 5% CO_2_. 

The cell viability was determined by using MTS from Promega. The cells (1 × 10^4^ cells per well) were seeding in 96-well plates in 100 µL of medium and were incubated for 24 h. Then, the cells were treated with various concentrations of the investigated samples, positive control (etoposide, 30 µg/mL) or vehicle (0.4% DMSO in culture medium) and incubated for 24 h. Subsequently, 20 µL of MTS was added at 20 h of treatment for 4 h, after that, the absorbance at 490 nm was measured using a microplate reader (Biotek, Winooski, VT, USA). The absorbance of control cells (untreated) was taken as 100% viability. The concentration of DMSO together with the treatments, in the culture medium was ≤ 0.4%, *v*/*v*.

### 3.7. Measurement of Nitrite Concentration

RAW 264.7 cells were seeding (2 × 10^4^ cells in 200 µL) and incubated for 24 h, then, were treated with various concentrations of the investigated samples, indomethacin (30 µg/mL) or DMSO (0.4%, *v*/*v*) for 2 h followed by stimulation with 1 µg/mL of LPS (from *E*. *coli* purchased from Sigma Aldrich, St. Louis, MO, USA) for 21 h. The nitrite is a stable metabolite of the NO and its concentration was calculated with Griess reagent and the aid of a standard curve of NaNO_2_. Cell-free supernatants (50 µL) were collected and incubated for 10 min with the Griess reagent (50 µL of 0.1% *N*-(1-napthyl)-ethylenediamine dihydrochloride and 50 µL of 1% sulphanilamide both in 2.5% phosphoric acid) at room temperature and absorbance at 540 nm was measured using a microplate reader. 

### 3.8. TPA-Induced Ear Edema in Mice

Edema in mice ear was induced with TPA purchased from Sigma Aldrich (St. Louis, MO, USA), following the method previously described [28]. All animals were treated in accordance with Official Mexican Rule of Technical Specifications for the Production, Care and Use of Laboratory Animals (NOM-062-ZOO-1999 Guidelines) and international ethical guidelines for the care and use of experimental animals. The experimental protocol was authorized by the local Health Research Committee (Instituto Mexicano del Seguro Social (IMSS)). The experiments were carried out with male ICR mice weighing between 25–30 g (five animals per treatment). The dose evaluated for the extracts and indomethacin (positive control) was of 1.0 mg/ear. The treatments were applied topically on right ear and vehicle on left ear, thereafter, the edema was induced by administration topically of TPA (2.5 µg) over both ears. Six hours after administration of TPA, the animals were sacrificed by cervical dislocation. Circular sections 6 mm in diameter were taken from both the treated and non-treated ears and weighed to determine the inflammation.

### 3.9. Antiproliferative Assay

PC3 (prostate), HeLa (cervical) and Hep3B and HepG2 (hepatocellular) human cancer cell lines were purchased from ATCC (Manassas, VA, USA). We also included immortalized human hepatocyte cell line (IHH) as a control of non-cancerous cells [29]. PC3 was grown in RPMI-1640 medium (Sigma Aldrich, St. Louis, MO, USA), while Hep3B, HepG2, HeLa and IHH in DMEM medium (Invitrogen, Thermo Fisher Scientific, Waltham, MA, USA) supplemented with 10% SFB and 2 mM glutamine, all cultures were incubated at 37 °C in 5% CO_2_. 

Cells (4 × 10^3^ cells/well) were seeding in 96-well plates. The cells were treated with the investigated samples (at 0.01, 0.1, 1, 10 and 100 µg/mL) and incubated at 37 °C in 5% CO_2_ for 72 h. PTX was used as positive control. The number of viable cells in proliferation was checked by MTS assay, following the manufacturer’s instructions. The absorbance was measured at 450 nm using an automated ELISA reader (Promega, Madison, WI, USA).

### 3.10. Cell Cycle Analysis

PC3, Hep3B, HepG2 and HeLa cells (1.25 × 10^5^ cells/well) were seed in 6-well plates and allowed to attach overnight at 37 °C in 5% CO_2_. Exponential growing cells were treated with Compound **1** to the IC_50_ values for 72 h. PTX (10 nM) was included as a positive control of the G_2_/M phase arrest. Cells from each treatment were trypsinized and collected into single cell suspensions, centrifuged, and fixed in cold ethanol (70%) overnight at −20 °C. The cells were then treated with 0.01 M RNase (Sigma Aldrich, St. Louis, MO, USA) and stained with 7.5 µg/mL PI (Invitrogen) for 30 min in the dark. The percentage of cells in sub-G_1_, G_1_, S, and G_2_/M phases was analyzed with a flow cytometer (Becton Dickinson, FACS Calibur; Beckman Coulter, Inc., Brea, CA, USA) using 10,000 cells for each sample. Data obtained from the flow cytometer were analyzed using the FlowJo Software (Tree Star, Inc., Ashland, OR, USA).

### 3.11. Study of Cell Death by Annexin V-FITC/PI Staining 

Apoptosis was evaluated using Annexin V Apoptosis Detection Kit FITC (Thermo Fisher Scientific, CA, USA). Hep3B cells (2.5 × 10^5^ cells) were treated with **1** (21.31 µM). PTX (20 nM) was used as positive control of apoptosis. After 72 h, the cells were harvested and washed twice with ice-cold PBS (0.01 M, pH 7.2). After 5 min of centrifuging at 200 g. Annexin V-FITC/PI staining were performed according to manufacturer’s instruction. Cell apoptosis was analyzed on flow cytometer (10,000 cells for each sample). Data obtained from the flow cytometer were analyzed using the FlowJo Software.

### 3.12. Cell Morphology Observation

The morphological changes of Hep3B cells exposed to Compound **1** were evaluated using acridine orange (AO) and ethidium bromide (EB) staining. In brief, Hep3B cells (2 × 10^4^ cells per well) were seeding in 24-well plates and exposed with Compound **1** at 21.31 µM for 72 h. After 72 h of treatment cells were exposed to a solution of 100 µg/mL AO and 100 µg/mL EB, according to reported procedures [30]. For controls, the cells were grown 71 h before being treated 30 min with H_2_O_2_ (positive control of apoptosis) or being boiling in water at 95 °C for 10 s (positive control of necrosis). Then, the cells were observed using a fluorescence microscope. 

### 3.13. RT-PCR

First, 1.25 × 10^5^ Hep3B cells were plated and treated with **1** at 21.31 µM for 72 h, then total RNA was isolated employing a Quick-RNA MiniPrep Kit (Zymo Research, Irvine, CA, USA), following the manufacturer’s instructions. RNA was quantified using NanoDrop ND-1000 (Thermo Scientific, Waltham, MA, USA) and the RNA content was normalized. The RT-PCR was performed using a One-Step RT-PCR Kit with Thermo-Start Taq (Thermo Scientific, Waltham, MA, USA) following the manufacturer’s instructions. PCR was performed with the following primers sequences: Bcl-2 (F 5′-TAC AGG CTG GCT CAG GAC TAT-3′; R 5′-CGC AAC ATT TTG TAG CAC TCT G-3′), Bax (F 5′-CCC GAG AGG TCT TTT TCC GAG-3′; R 5′-CCA GCC CAT GAT GGT TCT GAT-3′), GAPDH (F 5′-CAA GGT CAT CCA TGA CAA CTT TG-3′; R 5′-GTC CAC CAC CCT GTT GCT GTA G-3′). Sequencing was performed at the Instituto de Biotecnología of Universidad Nacional Autónoma de México. The reaction products of the samples were analyzed in 1.5% agarose gel.

### 3.14. Caspase 3/7

Hep3B cells (8 × 10^3^ cells per well) were plated on 96-well plates and treated with **1** at 21.31 µM for 72 h. After treatment, the caspase 3/7 activity was determined using the luminescent Caspase-Glo^®^ 3/7 Assay (Promega, Madison, WI, USA) following the manufacturer’s instructions. The results were represented as relative units of luminescence.

### 3.15. Statistical Analysis 

Statistical calculations were subjected with the GraphPad Prism^®^ version 5.0 software (Graphpad Software Inc., La Jolla, CA, USA). Results are expressed as mean ± S.D. of at least three independent experiments each by triplicate. Unless otherwise specified, one-way analysis of variance (ANOVA) followed by Dunnett test were performed. *p* < 0.05 was significant.

## Figures and Tables

**Table 1:** ^1^H (500 MHz) and ^13^C^*^ (125 MHz) NMR data for Compound **1** in C_6_D_6_.

Position	δ_H_	δ_C_	Position	δ_H_	δ_C_
CH_3_-1	1.69	25.89	CH_2_-21	2.21–2.16	27.08
C-2		131.15*^a^*	CH-22	5.26	124.73
CH-3	5.25	125.00*^b^*	C-23		135.22*^e^*
CH_2_-4	2.22–2.16	27.29*^c^*	CH_2_-24	2.12	40.27*^d^*
CH_2_-5	2.12–2.08	40.27*^d^*	CH_2_-25	2.23–2.15	27.18*^g^*
C-6		135.05*^e^*	CH-26	5.30	124.82*^f^*
CH-7	5.30	124.86*^f^*	C-27		135.28*^e^*
CH_2_-8	2.23–2.15	27.30*^g^*	CH_2_-28	2.12–2.08	40.38*^d^*
CH_2_-9	2.12–2.08	40.23*^d^*	CH_2_-29	2.22–2.16	27.17*^c^*
C-10		135.09*^e^*	CH-30	5.25	124.98*^b^*
CH-11	5.26	124.54	C-31		131.17*^a^*
CH_2_-12	2.19–2.15	26.91	CH_3_-32	1.69	25.89
CH_2_-13	2.04	40.03	CH_3_-33	1.57	17.78
C-14		137.29	CH_3_-34	1.62	16.45*^h^*
CH-15	5.36	129.04	CH_3_-35	1.60	16.18*^h^*
CH-16	4.39	68.62	CH_3_-36	1.61	16.76
CH_2_-17	2.40, 2.26	37.13	CH_3_-37	1.59	16.76
CH-18	5.34	120.92	CH_3_-38	1.60	16.18*^h^*
C-19		137.87	CH_3_-39	1.62	16.45*^h^*
CH_2_-20	2.11–2.09	40.25*^d^*	CH_3_-40	1.57	17.78

^*^ Resonances of ^13^C were obtained by using DEPTQ experiment. *^a–h^* The values of the resonances with same letters could be exchangable among them, since that cannot be unambiguously established due to overlapping of crosspeaks in HSQC and HMBC spectra.

**Table 2:** Maximal inhibitory concentration IC_50_ values of extracts (µg/mL) and Compounds **1**–**3** (µM) against various cancer cell lines and one non-cancerous (IHH).

Treatment	HeLa	PC3	HepG2	Hep3B	IHH
Th-H	N.D.*^b^*	N.D.	21.6 ± 1.9	32.7 ± 0.9	77 ± 7
Th-D	N.D.	N.D.	33.6 ± 2.2	> 100	> 100
Th-HA	N.D.	N.D.	31 ± 1.4	36.1 ± 1.5	84 ± 10
1	37.3 ± 3.5	32.0 ± 4.4	26.6 ± 3.5	21.3 ± 3.5	88.8 ± 8.9
2	95.7 ± 12.0	74.2 ± 5.0	76.6 ± 6.0	50.3 ± 5.9	76.6 ± 6.0
3	84.1 ± 8.9	76.4 ± 6.6	74.2 ± 5.5	69.7 ± 5.5	74.1 ± 5.5
Paclitaxel*^a^*	0.01 ± 0.002	0.0154 ± 0.0035	0.0065 ± 0.0007	0.008 ± 0.001	0.08 ± 0.005

*^a^* IC_50_ (µM) values of positive control; *^b^* N.D. (Non-determinate).

## Supplementary Materials

The following are available online. Effect of extracts and fractions of *T. hirsutissima* on cell viability and NO production in RAW 264.7 cells, Chromatogram of F4-2-2, 1D and 2D NMR spectra of **1**, ^1^H NMR spectra of 1a and 1b and effect of 1 on cell cycle of Hep3B, HepG2, PC3 and HeLa cells.
