# Supplementary material for: 16-Hydroxy-Lycopersene, a Polyisoprenoid Alcohol Isolated from Tournefortia hirsutissima, Inhibits Nitric Oxide Production in RAW 264.7 Cells and Induces Apoptosis in Hep3B Cells
Source: Molecules. 2019 Jun 26;24(13):2366. doi: 10.3390/molecules24132366 (PMC6651038; doi:10.3390/molecules24132366)
Supplement: Supplementary file 1 [file molecules-24-02366-s001.pdf]

SUPPLEMENTARY MATERIAL

16-Hydroxy-Lycopersene, a Polyisoprenoid Alcohol Isolated from *Tournefortia hirsutissima*, Inhibits Nitric Oxide Production in RAW 264.7 Cells and Induces Apoptosis in Hep3B Cells

Israel Hurtado-Díaz [1], Jessica Nayelli Sánchez-Carranza [2], Antonio Romero-Estrada [1], Leticia González-Maya [2], Judith González-Christen [2], Maribel Herrera-Ruiz [3] and Laura Alvarez [1,*]

[1] Centro de Investigaciones Químicas-IICBA, Universidad Autónoma del Estado de Morelos, Cuernavaca 62209, Morelos, México; ihurtado@uaem.mx (I.H.-D.); are@uaem.mx (A.R.-E.)

[2] Facultad de Farmacia, Universidad Autónoma del Estado de Morelos, Cuernavaca 62209, Morelos, México; jessica.sanchez@uaem.mx (J.N.S.-C.); letymaya@uaem.mx (L.G.-M.); judith.gonzalez@uaem.mx (J.G.-C.)

[3] Centro de Investigación Biomédica del Sur, Instituto Mexicano del Seguro Social, Xochitepec 62790, Morelos, México; cibis_herj@yahoo.com.mx

* Correspondence: lalvarez@uaem.mx; Phone/Fax: +52-777-329-7997

List of content

Figure S1. Effect of extracts of n-hexane (Th-H), dicloromethane (Th-D) and hydroalcoholic (Th-D) from leaves of *Tournefortia hirsutissima* at 1 mg/ear dose on the edema in mice ear induced by TPA.

Figure S2. Inhibition of the NO production in LPS-stimulated RAW 264.7 cells by Th-H, Th-D, Th-HA, F4, F4-1, F4-2, and F4-4 to indicated doses.

Figure S3. Effect of Th-H, Th-D, Th-HA, F4, F5, F4-1, F4-2, and F4-4 to indicated doses on cell viability of RAW 264.7 cells by using MTS assay.

Figure S4. Analysis of F4-2-1 by using GC/MS. The most abundant component in this fraction was bis (2-ethylhexyl) phthalate with a percentage of 55.84%.

Figure S5. Chromatogram of F4-2-2 by using analytical HPLC, monitored at 210 nm using Merck column (Performance RP-18e, 100 x 4.6 mm).

Figure S6. ^{1}H NMR spectrum (500 MHz, benzene-d_6) of compound **1**.

Figure S7. ^{1}H NMR spectrum expansions (500 MHz, benzene-d_6) of compound **1**.

Figure S8. DEPTQ NMR spectrum (125 MHz, benzene-d_6) of compound **1**.

Figure S9. ^{1}H - ^{1}H COSY spectrum (benzene-d_6) of compound **1**.

Figure S10. TOCSY spectrum (benzene-d_6) of compound **1**.

Figure S11. HSQC spectrum (benzene-d_6) of compound **1**.

Figure S12. HMBC spectrum (benzene-d_6) of compound **1**.

Figure S13. ^{1}H NMR spectrum (500 MHz, pyridine-d_5) of (*S*)-MTPA ester of **1** (**1a**).

Figure S14. ^{1}H NMR spectrum (500 MHz, pyridine-d_5) of (*R*)-MTPA ester of **1** (**1b**).

Figure S15. The cell cycle of (A) Hep3B, (B) HepG2, (C) PC3 and (D) HeLa cells by flow cytometry treated with **1** to its CI_{50} and PTX 10 nM.

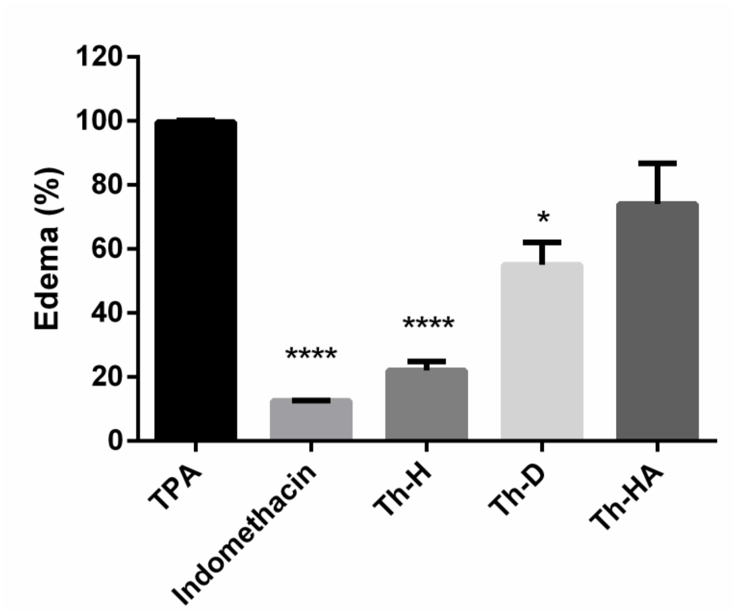

Figure S1. Effect of extracts of n-hexane (Th-H), dicloromethane (Th-D) and hydroalcoholic (Th-D) from leaves of *Tournefortia hirsutissima* at 1 mg/ear dose on the edema in mice ear induced by TPA. Indomethacin (1 mg/ear) was used as control. * $P < 0.05$, ** $P < 0.01$, *** $P < 0.001$, **** $P < 0.0001$ compared to the TPA control.

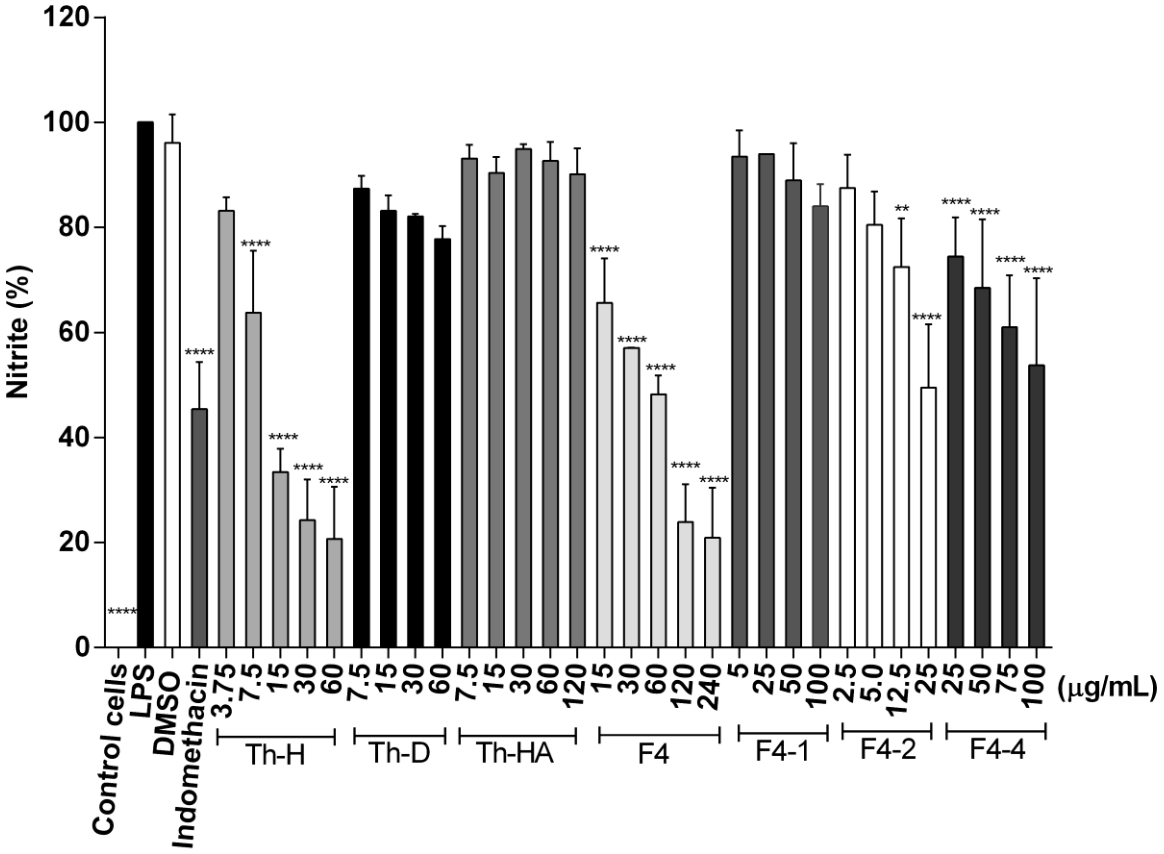

Figure S2. Inhibition of the NO production in LPS-stimulated RAW 264.7 cells by Th-H, Th-D, Th-HA, F4, F4-1, F4-2, and F4-4 to indicated concentrations. Cells were treated with the investigated samples, DMSO (0.4%, *v*/*v*) or indomethacin (30 μg/mL) 2 h before stimulation with LPS (1.0 μg/mL). The nitrite concentration was determined by Griess

method and is expressed in percentage. All data represent the mean ± standard deviation of at least three independent experiments performed by triplicate. Statistical significance was determined by one-way ANOVA followed by Dunnett's test. * $P < 0.05$, ** $P < 0.01$, *** $P < 0.001$ and **** $P < 0.0001$ compared with LPS group.

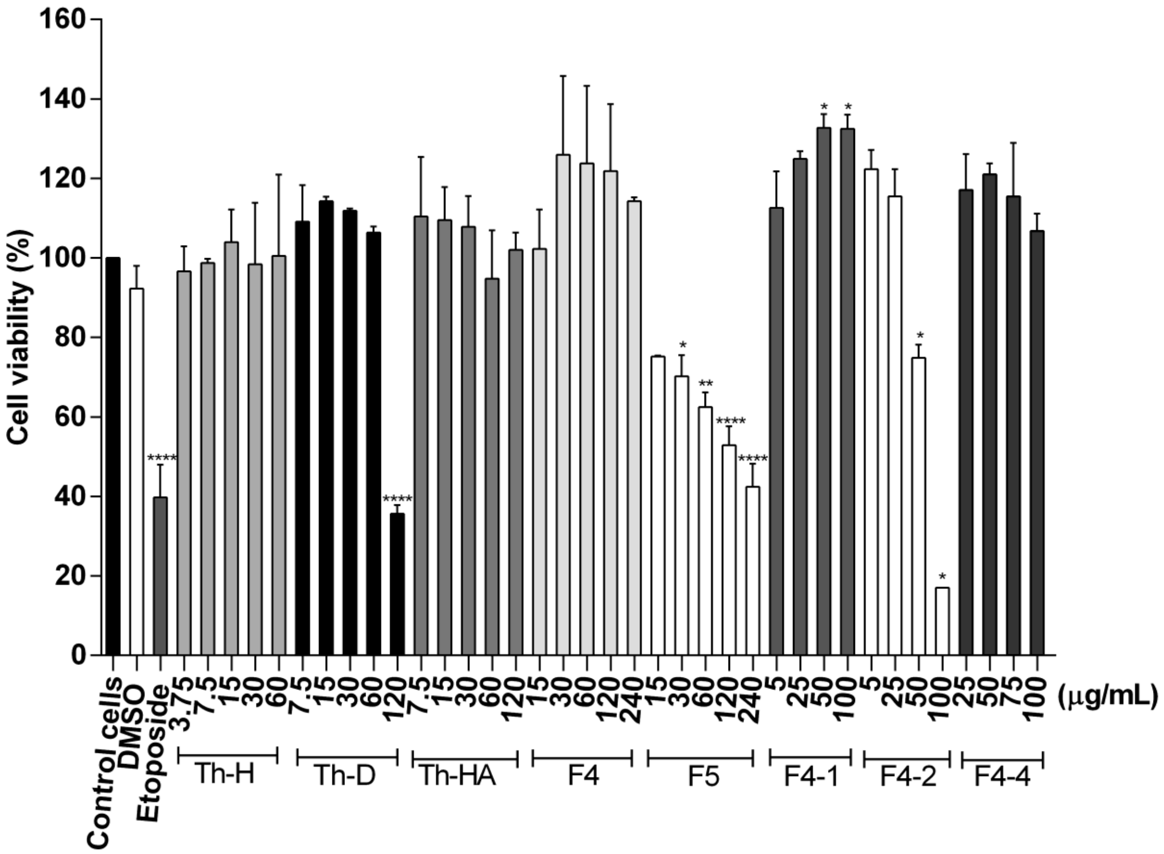

Figure S3. Effect of Th-H, Th-D, Th-HA, F4, F5, F4-1, F4-2, and F4-4 to indicated concentrations on cell viability of RAW 264.7 cells by using MTS assay. Cell viability is expressed in percentage. All data represent the mean ± standard deviation of at least three independent experiments performed by triplicate. Statistical significance was

determined by one-way ANOVA followed by Dunnett's test. * $P < 0.05$, ** $P < 0.01$, *** $P < 0.001$ and **** $P < 0.0001$ compared with control cells group (without treatment).

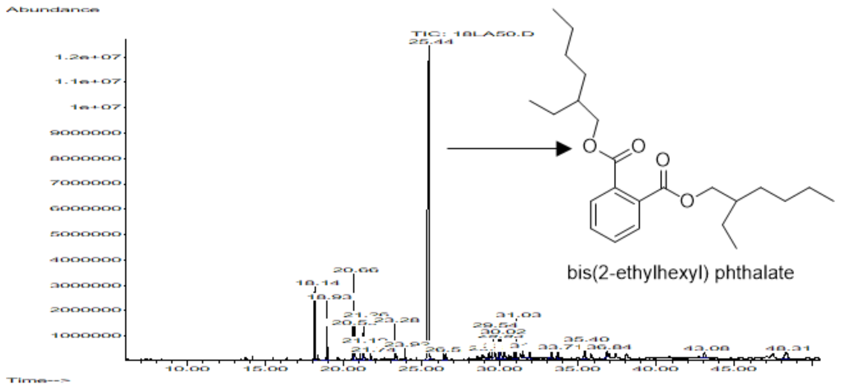

Figure S4. Analysis of F4-2-1 by using GC/MS. The most abundant component in this fraction was bis (2-ethylhexyl) phthalate with a percentage of 55.84%.

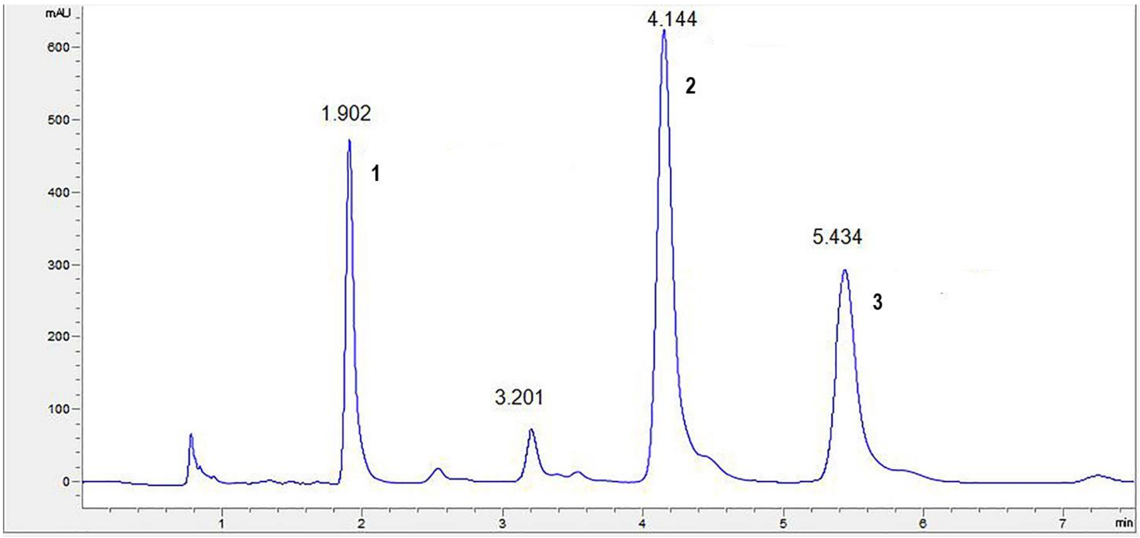

Figure S5. Chromatogram of F4-2-2 by using analytical HPLC, monitored at 210 nm using Merck column (Performance RP-18e, 100 x 4.6 mm). The mobile phase consisted of a gradient of iPrOH : MeOH : H_2O (30 : 67 : 03 to 30 : 70 : 00, *v*/*v*). Flow rate was 2.0 mL/min and sample injection of 7 µL (1.5 mg/mL). The peaks with retention times at 1.90, 3.20 and 4.14 min correspond to **1**, **2** and **3**, respectively.

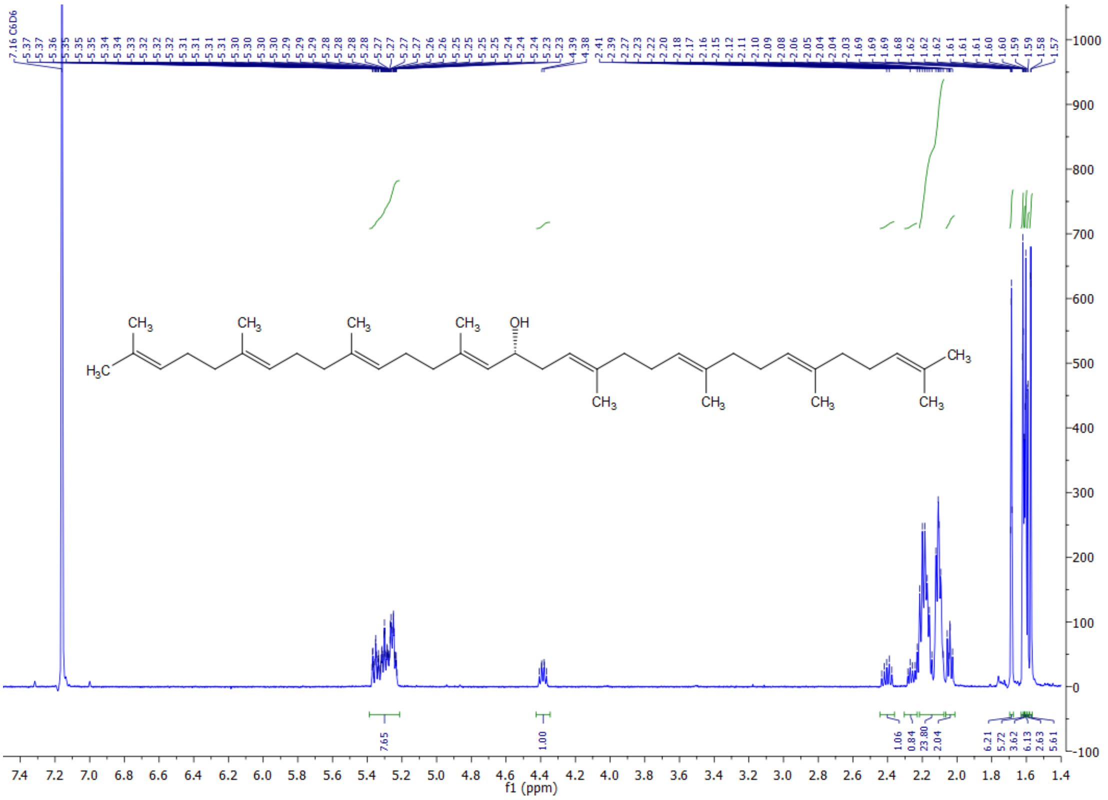

Figure S6. ^{1}H NMR spectrum (500 MHz, benzene-d_6) of compound **1**.

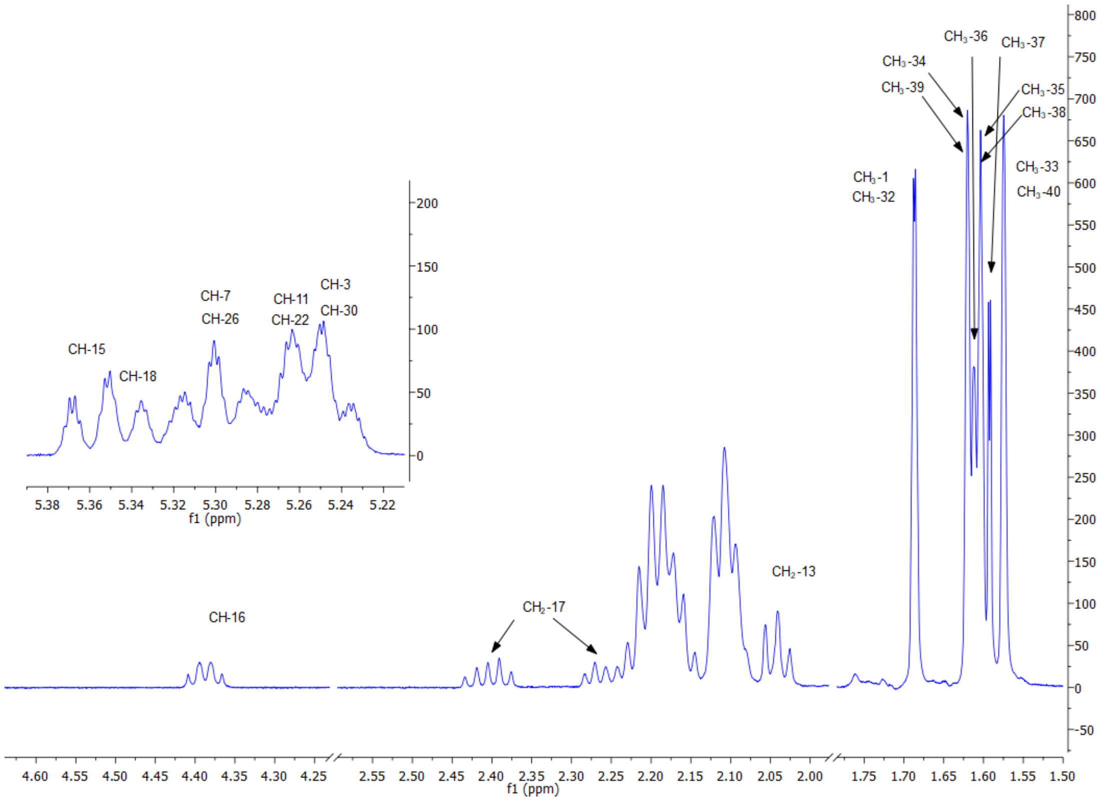

Figure S7. ^{1}H NMR spectrum expansions (500 MHz, benzene-d_6) of compound **1**.

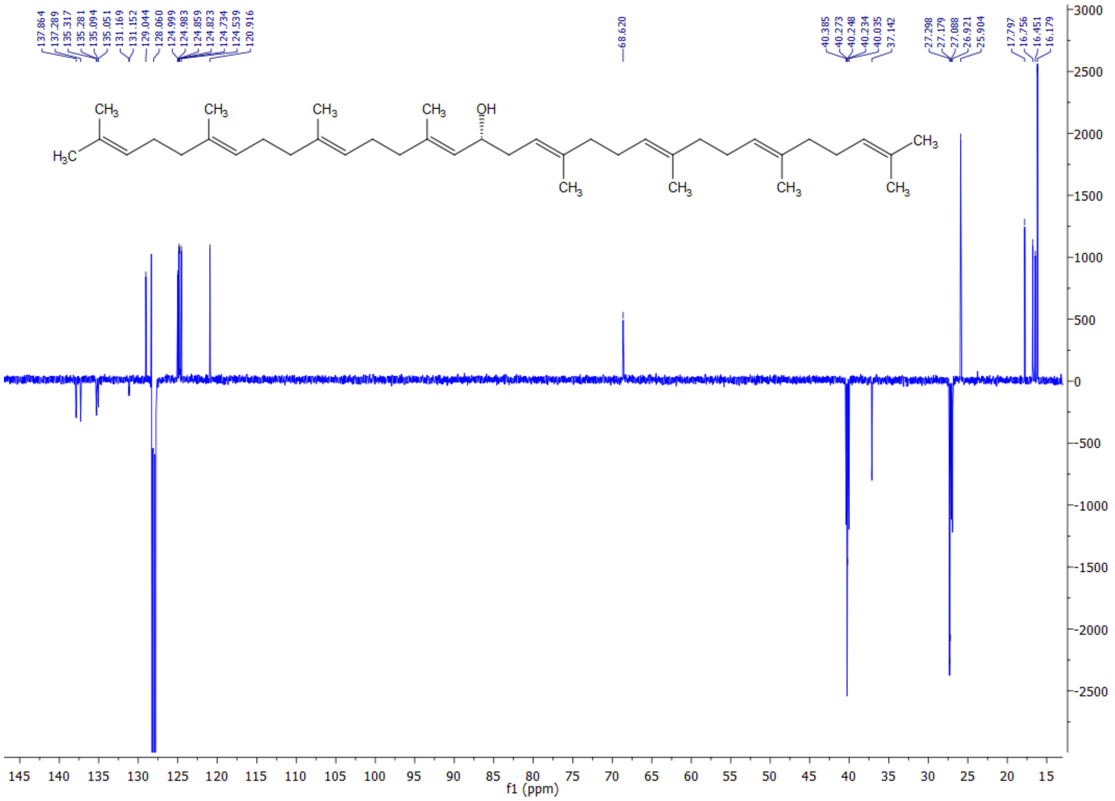

Figure S8. DEPTQ NMR spectrum (125 MHz, benzene-d_6) of compound **1**.

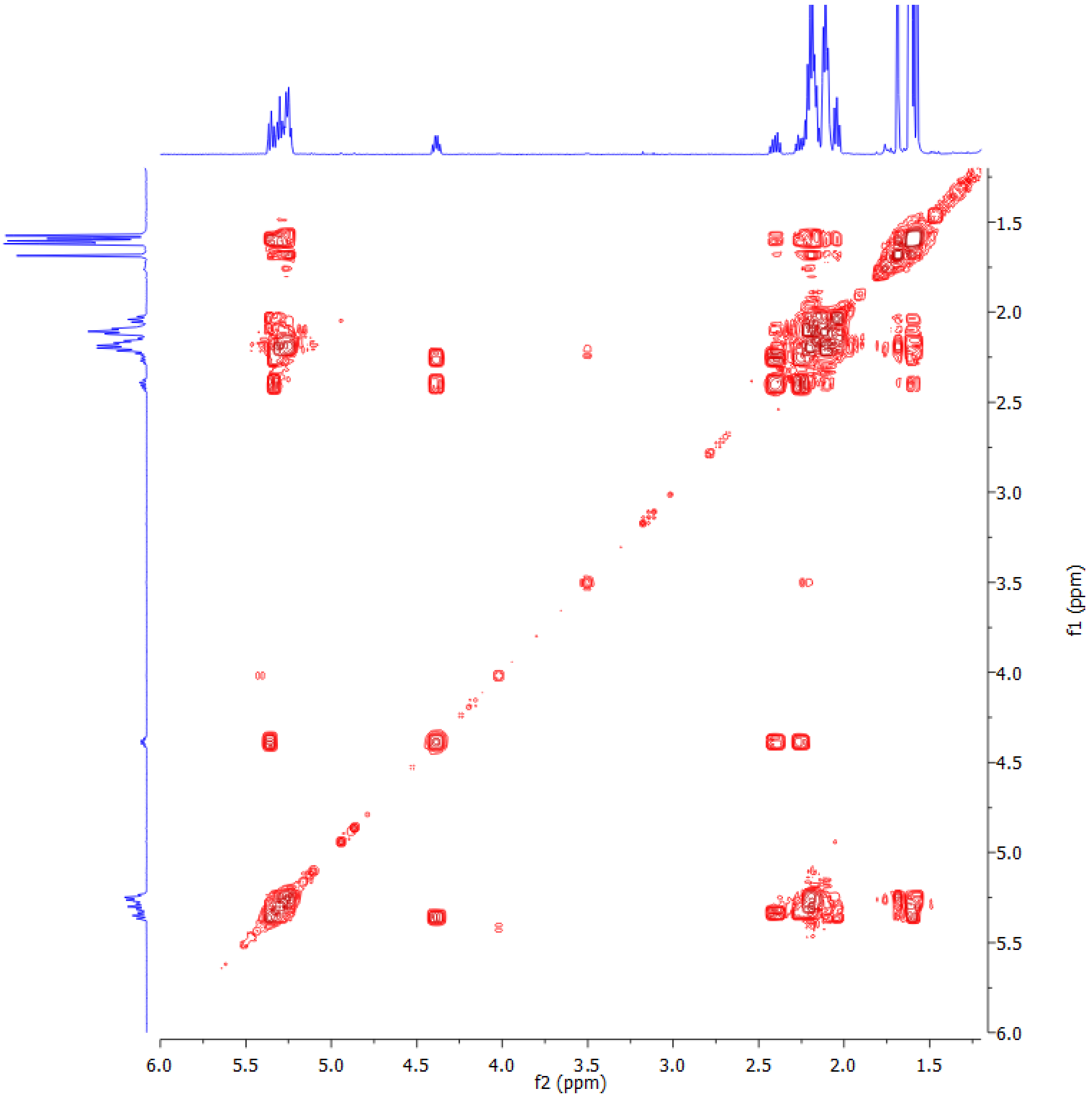

Figure S9. ^1H - ^1H COSY spectrum (benzene-d_6) of compound **1**.

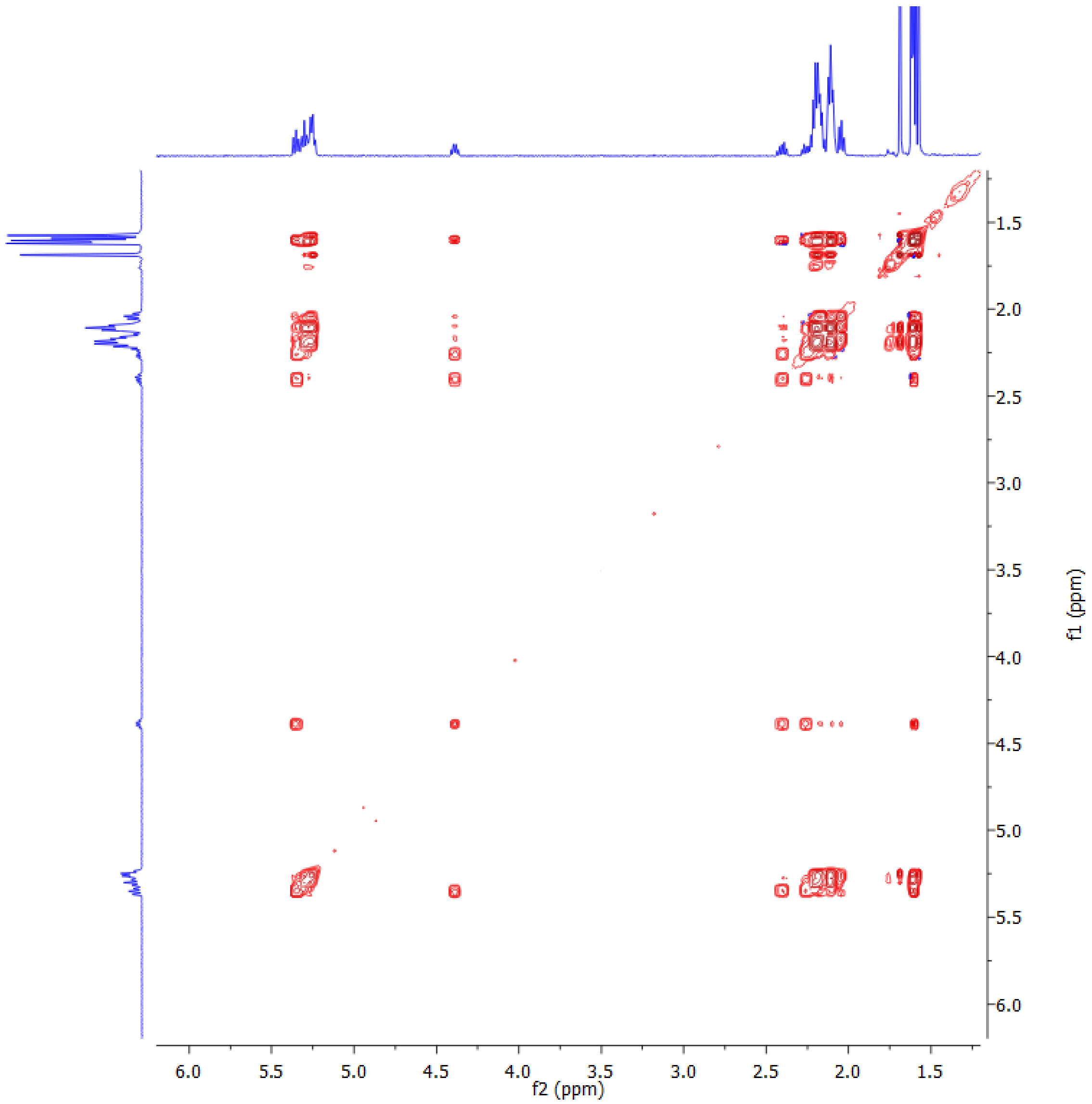

Figure S10. TOCSY spectrum (benzene-d_6) of compound **1**.

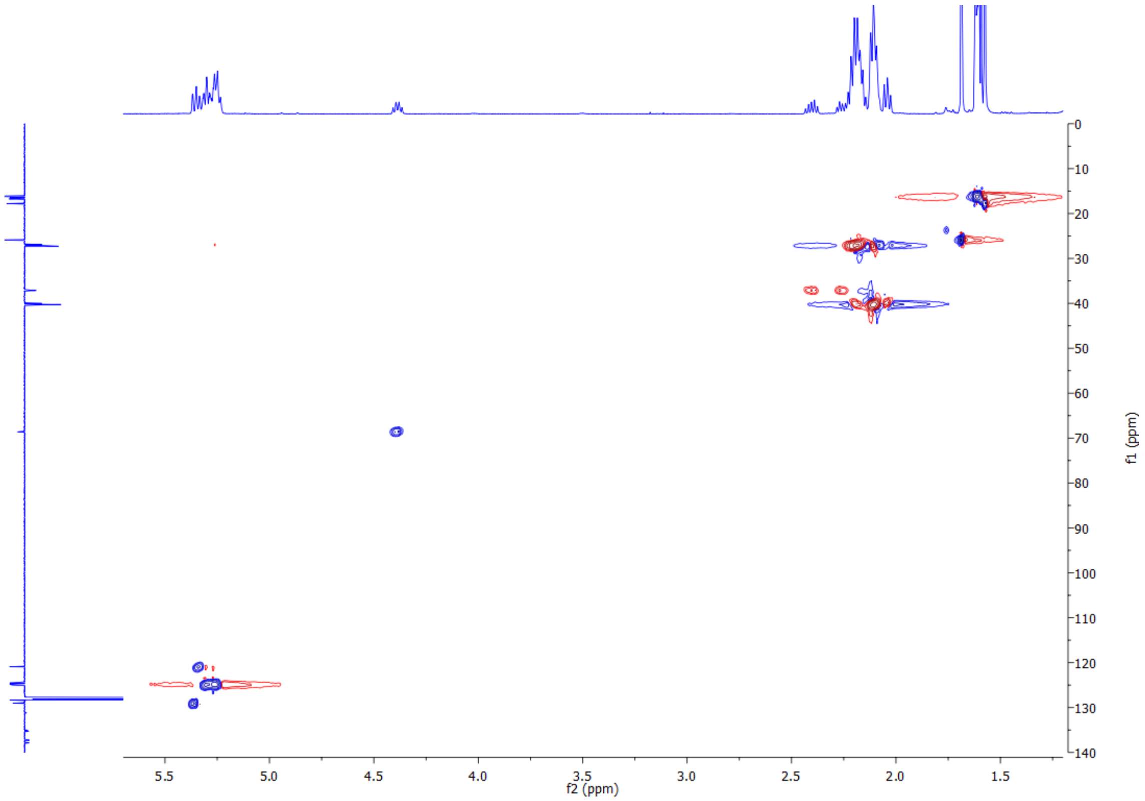

Figure S11. HSQC spectrum (benzene-d_6) of compound 1.

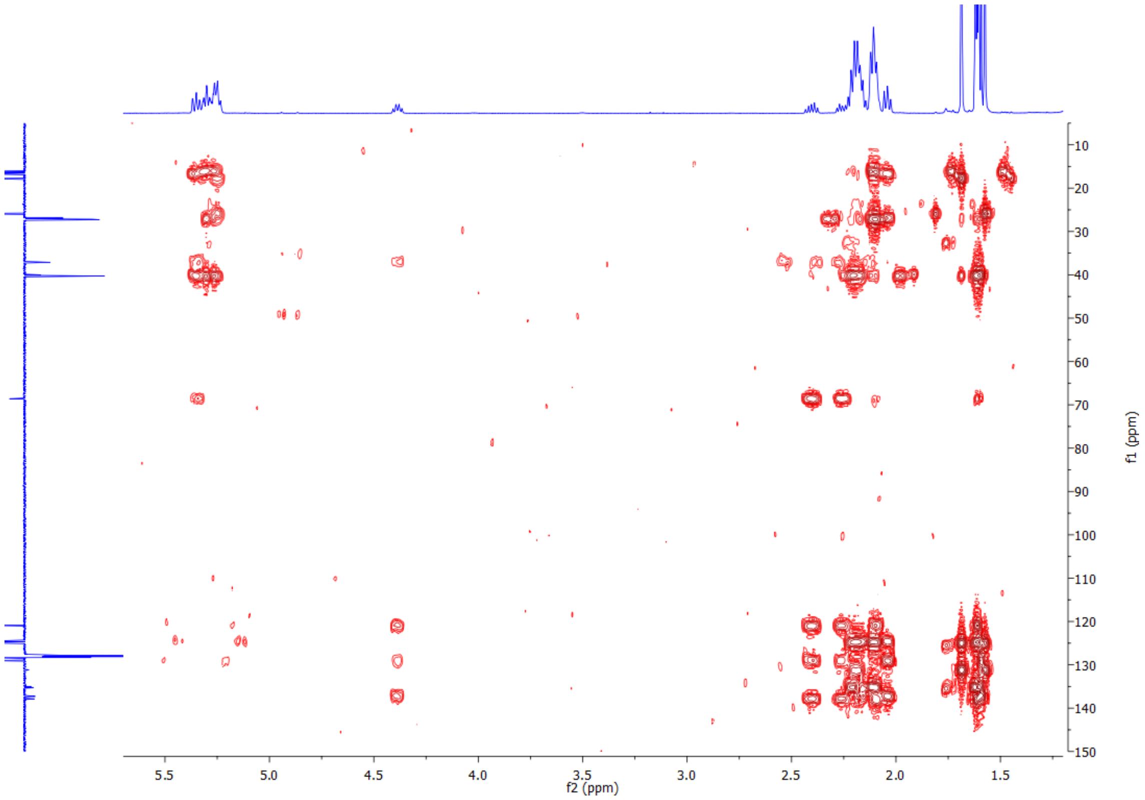

Figure S12. HMBC spectrum (benzene-d_6) of compound **1**.

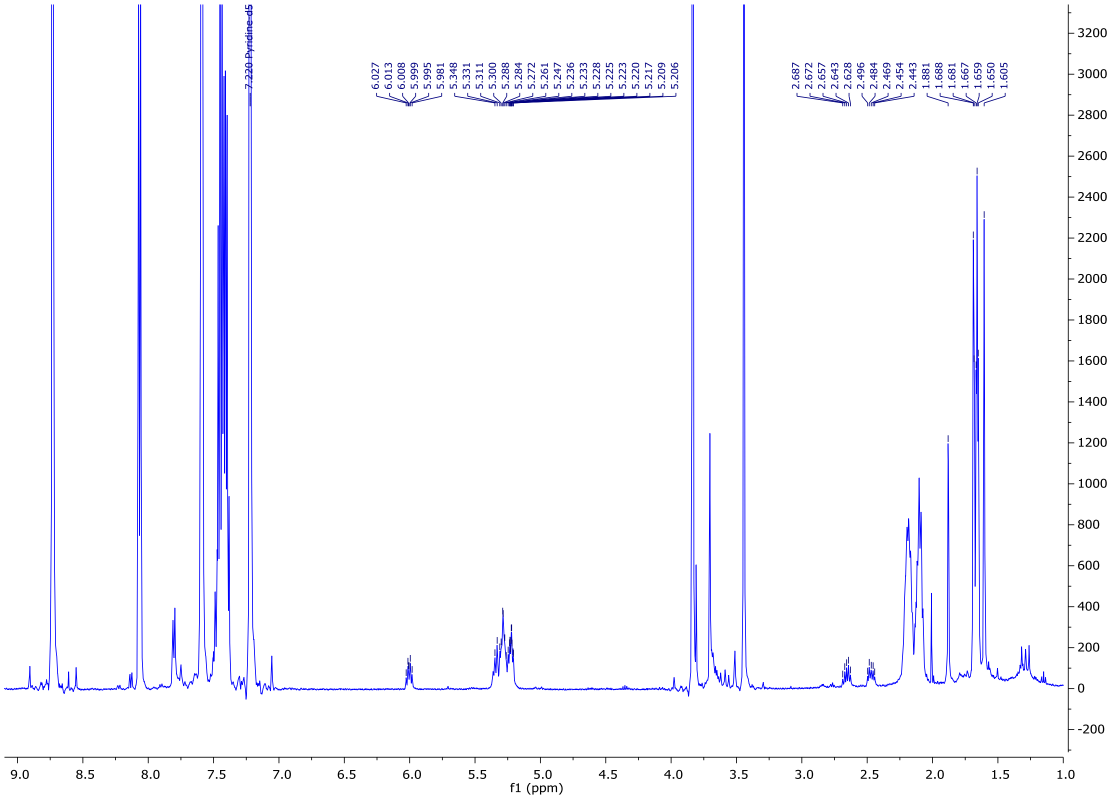

Figure S13. ^{1}H NMR spectrum (500 MHz, pyridine-d_5) of (*S*)-MTPA ester of **1** (**1a**).

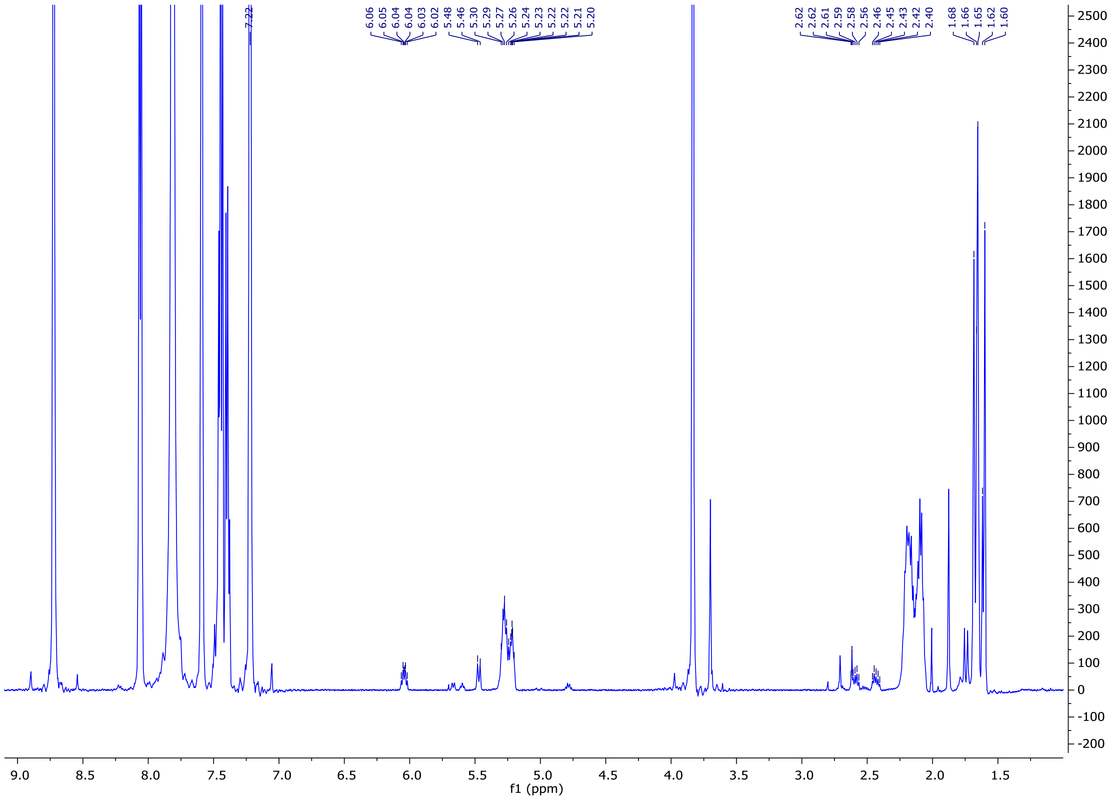

Figure S14. ^{1}H NMR spectrum (500 MHz, pyridine-d_5) of (*R*)-MTPA ester of **1** (**1b**).

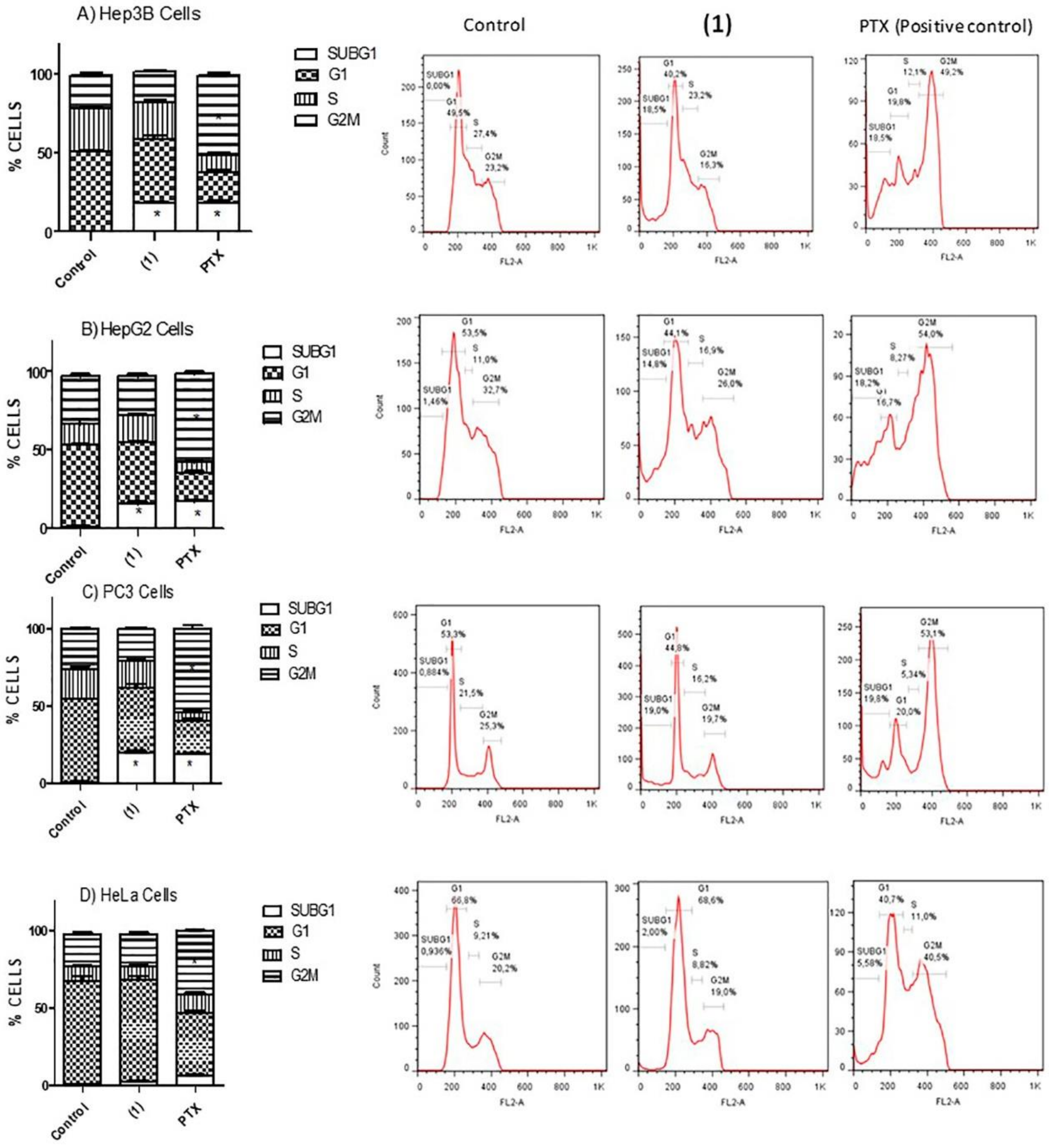

Figure S15. The cell cycle of (A) Hep3B, (B) HepG2, (C) PC3 and (D) HeLa cells by flow cytometry treated with **1** to its IC_{50} and PTX 10 nM. The data are the means ± D.E. of three independent experiments. Statistical significance was determined by one-way ANOVA followed by Dunnett's test. * $P < 0.05$ compared to the non-treated control.
